# Primary angiitis of the central nervous system presenting with subacute and fatal course of disease: a case report

**DOI:** 10.1186/1471-2377-5-16

**Published:** 2005-09-14

**Authors:** Carsten Lukas, Kathy Keyvani, Christian Börnke

**Affiliations:** 1 Department of Neurology, St. Josef-Hospital, Ruhr-University Bochum, Gudrunstr. 56, 44791 Bochum, Germany; 2 Institute of Neuropathology, Westfälische Wilhelms-University, Domagkstr. 19, 48149 Münster, Germany

## Abstract

**Background:**

Primary angiitis of the central nervous system is an idiopathic disorder characterized by vasculitis within the dural confines. The clinical presentation shows a wide variation and the course and the duration of disease are heterogeneous. This rare but treatable disease provides a diagnostic challenge owing to the lack of pathognomonic tests and the necessity of a histological confirmation.

**Case presentation:**

A 28-year-old patient presenting with headache and fluctuating signs of encephalopathy was treated on the assumption of viral meningoencephalitis. The course of the disease led to his death 10 days after hospital admission. Postmortem examination revealed primary angiitis of the central nervous system.

**Conclusion:**

Primary angiitis of the central nervous system should always be taken into consideration when suspected infectious inflammation of the central nervous system does not respond to treatment adequately. In order to confirm the diagnosis with the consequence of a modified therapy angiography and combined leptomeningeal and brain biopsy should be considered immediately.

## Background

Primary angiitis of the central nervous system (PACNS) is an idiopathic, recurrent vasculitis confined to the central nervous system and the dural reflections. It may involve small and medium-sized leptomeningeal, cortical and subcortical arteries. The most common presentation is headache with encephalopathy accompanied by multifocal symptoms [1]. PACNS can occur at any age but there is a predominant appearance in the fourth to sixth decade [2]. The course and the duration of this disease show a wide variation. We describe a case with rapid progression and fatal outcome.

## Case presentation

**Figure 1:**
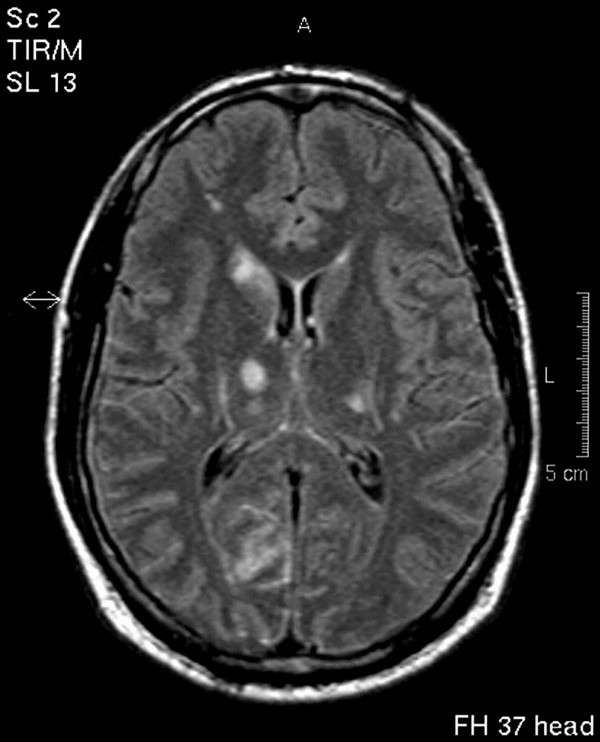
Cranial MRI (FLAIR) on the day of admission showing multiple lesions of the supratentorial grey and white matter.

A 28-year-old man complaining of acute hoarseness and tingling in his left upper and lower extremities was admitted to hospital. His past medical history revealed persistent frontal headache for the last 3 weeks. On admission the patient was nervous and slightly confused. Fever was not present, although the patient complained of flu-like symptoms 4 weeks before. On neurologic examination dysarthria, a left facial weakness, clumsiness in rapid alternating movements of the left hand and dysmetric finger-to-nose testing beside abnormalities in pain and temperature sensation on the left side were found. Cranial computed tomography (CCT) scan was normal. Routine laboratory examinations showed no abnormalities. Serum antibodies to neurotropic viruses, HIV, Treponema pallidum and Borrelia burgdorferi were not found. The cerebrospinal fluid (CSF) contained inflammatory cells (32 lymphocytes/μl) and total CSF protein was elevated (930 mg/l). The glucose ratio was normal. Immunological studies and polymerase chain reactions for herpes simplex virus type 1, 2 and varicella-zoster virus were unobtrusive. Suspecting acute viral meningoencephalitis the patient was treated with aciclovir 3 × 750 mg/d. Within 24 hours after acute onset of focal neurologic symptoms remission of dysarthria was observed while the abnormal sensation and mild agitation were still present. Magnetic resonance (MR) scans performed 24 hours after acute onset of focal symptoms showed disseminated hyperintensities of the supratentorial grey and white matter with predominance in the right occipital region lacking enhancement after GdDTPA-injection (Figure 1). The MR-angiography was normal. The characteristics and location of these findings were not typical for acute disseminated encephalomyelitis or herpes simplex encephalitis. The follow-up MR scans showed an increase of white matter hyperintensities with enhancement after GdDTPA-injection. Repeated CSF analyses revealed no new aspects. During the hospital course only subfebrile body temperatures could be registered. Eight days after admission conventional angiography was considered but the patient developed severe disorientation and agitation. Within 12 hours he became unconscious and developed signs of transtentorial herniation. The emergency CCT-scan revealed massive generalized brain edema. Artificial ventilation was necessary. The patient was treated with steroids 2 × 1000 mg/d, but died 10 days after onset of his focal symptoms with signs of extensive brain stem dysfunction.

### Autopsy findings

**Figure 2:**
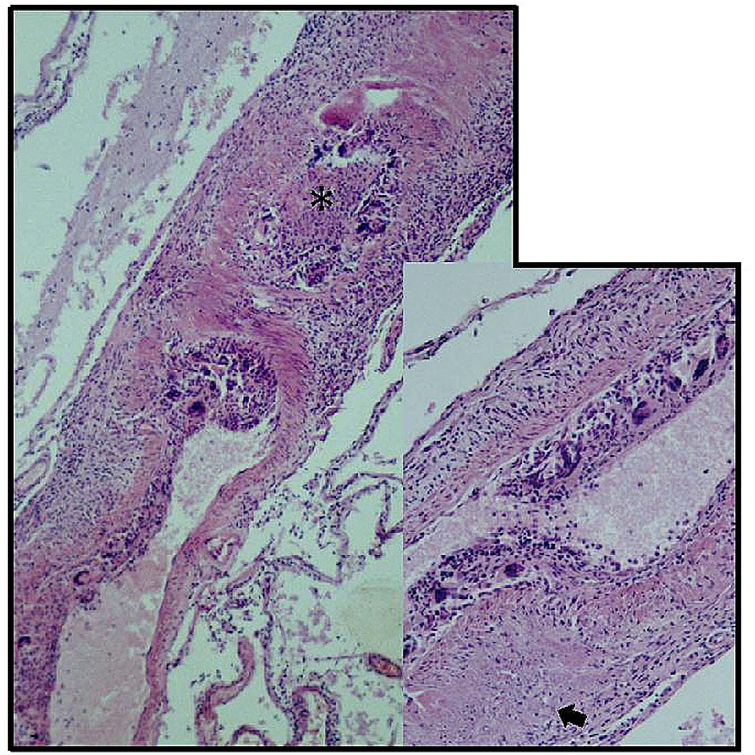
Leptomeningeal arteries show severe transmural inflammation with lumen obliteration (asterisk). The arrow indicates a fibrinoid necrosis in the vessel wall.

The postmortem examination revealed primary angiitis of the central nervous system (PACNS) with focal granulomatous inflammation and partially thrombotic occlusion of leptomeningeal and cerebral arteries (Figure 2) resulting in multiple acute infarction areas in the cerebrum. There was also a brisk acute hypoxic-ischemic encephalopathy with diffuse eosinophilic neuronal injury (particularly in the cerebral cortex, hippocampus, Purkinje cell layer, and olivary nucleus) leading to and sustaining a prominent swelling of the brain with terminal herniation and necrosis of the parahippocampal gyrus as well as tonsillar tissue. There was no deposition of amyloid beta protein (Abeta) in the brain parenchyma and vessel walls as revealed by immunohistochemistry for Abeta (6F/3D anti-Aβ monoclonal antibody to residues 8 – 17, dilution1:100, Dako). Outside the dural confines no signs of vasculitis or systemic disease were found.

## Conclusion

The clinical course of PACNS shows a wide variation, progressive courses with fatal outcome as well as benign courses have been reported. In general, the course of the untreated disease is, if clinical manifestation is protean, progressive, with death occurring within 9–12 months [2]. Shukla et al. reported on a patient with PACNS and an unusual prolonged clinical course of 8.5 years without treatment [3]. The ante-mortem diagnosis of PACNS depends on a combination of clinical, imaging and histological findings. Major differential diagnosis of PACNS include secondary vasculitides of the CNS which can occur in collagen vascular disorders, viral and bacterial infections, in malignancies and due to drugs and substance abuse [1,4]. Abeta-related angiitis (ABRA) that occasionally complicates cerebral amyloid angiopathy may also be taken into consideration, however, the mean age of presentation of ABRA seems to be higher than that of PACNS [5]. Young women with a history of headaches may present with benign angiopathy of the central nervous system [6,7]. Headache and encephalopathy are also common clinical features of posterior leukoencephalopathy. This newly recognised neurological disorder is usually seen in patients with uncontrolled hypertension, eclampsia and in patients treated with immunosuppressive drugs [8]. As recently published, posterior leukoencephalopathy can be associated with isolated CNS angiitis [9].

Examination of the CSF in PACNS is abnormal in 80–90% of patients and consists of lymphocytic pleocytosis and elevated protein [6]. MRI findings in PACNS are variable and non-specific. The most common findings are multiple bilateral asymmetrical supratentorial lesions, involving grey and white matter [10]. In a few cases mass lesions have been described [10,11], but nevertheless normal MRI and biopsy findings consistent with CNS vasculitis have also been found [12]. Abnormal conventional angiography may be useful to assume cerebral vasculitis [12,13], but many cases of PACNS have normal arteriograms while in cases with abnormal findings, such as multiple segmental narrowings of cerebral arteries, the diagnosis of PACNS could not be confirmed [14]. Moore has suggested criteria for the diagnosis of PACNS [2]. To fulfill these criteria systemic infections and inflammatory diseases have to be excluded. Combined leptomeningeal and brain biopsy has to confirm the presence of vascular inflammation. Biopsy should be performed prior to immunosuppressive treatment in order to exclude lesions of infectious, neoplastic or non-inflammatory etiology and to confirm a diagnosis of vasculitis. Due to the segmental nature of the lesions false-negative results can be obtained in up to 35% of biopsies [15-18]. The best established therapy is a combination of high-dose steroids with cyclophosphamide [19]. After clinical stabilization of the patient, treatment should be continued for at least 1 year [20].

In our case the duration between onset of neurological symptoms and death was markedly short. Fever, that one would expect in infectious inflammation of the CNS, was not present in our patient. Nevertheless, the lack of fever is not specific enough to guide diagnostic efforts. These conditions highlight the importance of early angiography and biopsy in cases of suspected vasculitis. Unfortunately, in our case the brief and fluctuating course of the disease with final deterioration within 10 days did not lead to angiography and brain biopsy in time. In conclusion even in young patients PACNS should always be taken into consideration especially when suspected infectious inflammation of the CNS does not respond to treatment adequately. In the face of a progressive clinical course and no specific etiological diagnosis one should not continue to treat CNS inflammatory disease empirically. Since PACNS would require a modification of the therapy, angiography and a combined leptomeningeal and cortical biopsy should be performed immediately.

## Competing interests

The author(s) declare that they have no competing interests.

## Authors' contributions

CL carried out the neurological examination, study of literature and substantial contributions in writing and design of the manuscript. CL prepared Figure 1. KK carried out the neuropathological investigations, description of this investigation and prepared Figure 2. CB reviewed and corrected the manuscript and gave final approval of the version to be published.

## Pre-publication history

The pre-publication history for this paper can be accessed here:


